# Advanced body composition assessment: from body mass index to body composition profiling

**DOI:** 10.1136/jim-2018-000722

**Published:** 2018-03-25

**Authors:** Magnus Borga, Janne West, Jimmy D Bell, Nicholas C Harvey, Thobias Romu, Steven B Heymsfield, Olof Dahlqvist Leinhard

**Affiliations:** 1 Department of Biomedical Engineering, Linköping University, Linköping, Sweden; 2 Center for Medical Image Science and Visualization (CMIV), Linköping University, Linköping, Sweden; 3 Advanced MR Analytics AB, Linköping, Sweden; 4 Department of Medical and Health Sciences, Linköping University, Linköping, Sweden; 5 Research Centre for Optimal Health, University of Westminster, London, UK; 6 MRC Lifecourse Epidemiology Unit, University of Southampton, Southampton, UK; 7 NIHR Southampton Biomedical Research Centre, University of Southampton, University Hospital Southampton NHS Foundation Trust, Southampton, UK; 8 Pennington Biomedical Research Center, Baton Rouge, Louisiana, USA

**Keywords:** body composition, magnetic resonance imaging

## Abstract

This paper gives a brief overview of common non-invasive techniques for body composition analysis and a more in-depth review of a body composition assessment method based on fat-referenced quantitative MRI. Earlier published studies of this method are summarized, and a previously unpublished validation study, based on 4753 subjects from the UK Biobank imaging cohort, comparing the quantitative MRI method with dual-energy X-ray absorptiometry (DXA) is presented. For whole-body measurements of adipose tissue (AT) or fat and lean tissue (LT), DXA and quantitative MRIs show excellent agreement with linear correlation of 0.99 and 0.97, and coefficient of variation (CV) of 4.5 and 4.6 per cent for fat (computed from AT) and LT, respectively, but the agreement was found significantly lower for visceral adipose tissue, with a CV of >20 per cent. The additional ability of MRI to also measure muscle volumes, muscle AT infiltration and ectopic fat, in combination with rapid scanning protocols and efficient image analysis tools, makes quantitative MRI a powerful tool for advanced body composition assessment.

## INTRODUCTION

The human body—as well as the body of every other animal—is mainly composed of four molecular-level components: water, fat, proteins and minerals, usually in that order of decreasing amounts.^1^ The substance that has attracted the most attention, from laypeople to medical professionals, is fat. This is, of course, motivated by the well-established fact that an excessive amount of body fat is related to increased morbidity and mortality. But also because adipose tissue (AT) is, by far, the most varying compartment—between individuals, but also within an individual over time. The most widely used way to estimate body fat is the body mass index (BMI)—body weight normalized by height squared (kg/m^2^). Being a very simple and inexpensive method, it is the basis for WHO’s definition of overweight (25≤ BMI <30) and obesity (BMI ≥30). However, for a given BMI, the body fat percentage changes with age, and the rate of this change is different depending on sex, ethnicity and individual differences.^2^ And while BMI correlates with fat accumulation and metabolic health in large populations, it is insensitive to the actual distribution of body fat.^3^

When comparing methods for body composition analysis, it is important to distinguish fat (triglyceride) from AT,^4^ which contains approximately 80 per cent fat, the rest being water, protein and minerals.^5^ While most of the body fat is stored in AT, fat is also present in organs such as liver and skeletal muscle. Today, it is well known that the metabolic risk related to fat accumulation is strongly dependent on its distribution. Central obesity and, in particular, ectopic fat accumulation are important metabolic risk factors.^6–8^ Large amounts of visceral AT (VAT) are related to increased cardiac risk,^8 9^ type 2 diabetes,^10 11^ liver disease^12^ and cancer.^13 14^ High levels of liver fat increase the risk for liver disease and type 2 diabetes,^15^ and increased muscle fat has been associated with increased risk for insulin resistance and type 2 diabetes^16^ and reduced mobility.^17^ While there are other anthropometric measures, such as waist circumference and waist-to-hip ratio, which more strongly correlate with metabolic risk,^18 19^ it is now well recognized that BMI and other anthropometric surrogate measures are poor predictors for individual fat distribution and metabolic risk.^3 20 21^

Besides fat, acting as the body’s long-term energy storage, skeletal muscles are of great interest to study, and the balance between the energy-consuming muscles and the energy-storing fat compartments is, of course, highly relevant in order to understand the metabolic balance of the body. Cachexia, involuntary loss of body weight, usually with disproportionate muscle wasting, is a life-threatening condition, often related to the progression of an underlying serious disease (eg, cancer^22^). In cancer, cachexia is defined as weight loss of >5 per cent over 6 months, BMI <20 kg/m^2^ or appendicular muscle mass normalized by body height squared of <7.26 kg/m^2^ or 5.45 kg/m^2^ for males and females, respectively.^23^ Sarcopenia, which can be related to cachexia, but is also associated with aging, is often defined as reduced physical performance following loss of muscle mass, usually accompanied by increased fat infiltration of the muscles.^24^ When diagnosing sarcopenia, muscle strength tests combined with muscle volume measurements are needed.^25^ Furthermore, Willis *et al* showed that muscle pathology progression over 1 year could be detected by quantitative MRI but not by assessing muscle strength or function.^26^ These examples illustrate the need for more sophisticated body composition analysis tools that go beyond simple anthropometric measures.

Since the early part of the last century, scientists have tried to determine the body composition in different ways, with a wide range of different physical principles and devices, and using different models and assumptions. Today, local in vivo measurements of different fat depots and fat infiltration in organs can be made using tomographic imaging techniques such as CT and MRI that were not even invented when the first scientific studies on body composition were published. These techniques are now recognized as golden standard for body composition analysis.^25 27^

The purpose of this paper is to give a brief introduction to the most commonly used methods for body composition analysis and a review of an MRI-based body composition analysis technique, comparing its performance to other methods. This includes a previously unpublished validation study of the agreement between this method and dual-energy X-ray absorptiometry (DXA).

## TECHNOLOGY OVERVIEW

A number of different techniques for body composition assessment have been developed, from very simple indirect measures such as waist-to-hip ratio and calipers to sophisticated direct volumetric measurements based on three-dimensional imaging techniques. There are also a range of invasive or in vitro methods for body composition analysis such as inhalation or injection of water-accumulating or fat-accumulating agents, or dissection and chemical analysis of cadavers. This overview will, however, focus solely on non-invasive in vivo measurement techniques.

### Hydrostatic weighing (densitometry)

Hydrostatic weighing (underwater weighing), or densitometry, is based on Archimedes’ principle. The difference of the body weight in air and water is used to compute the body’s density. Assuming a two-component model with different densities for fat mass and fat-free mass and correcting for the air volume in the lungs, the total body fat percentage can be estimated. Obviously, this technique cannot give any measurements of the distribution of AT or lean tissue (LT).

### Air displacement plethysmography (ADP)

ADP is perhaps better known under its commercial brand name BOD POD (Life Measurement, Concord, California, USA). Similar to hydrostatic weighing, ADP measures the overall body density and hence total body fat and LT but not their distributions. By putting the body in an enclosed chamber and changing the chamber’s volume, the volume of the displaced air (ie, the volume of the body) can be determined from the changes in air pressure. Since ADP is based on the same two-component model as hydrostatic weighing, it is also affected by the same confounders, mainly variations in bone mineral content (BMC) and hydration. Due to the limitations of the two-component model used in densitometry and ADP, a four-component (4C) model is often recommended.^28 29^ In addition to fat and LT, the 4C model also takes BMC and total body water (TBW) into account. However, these two additional components have to be measured by other techniques (eg, DXA for the BMC and deuterium oxide dilution for TBW^30^) The repeatability (coefficient of variation (CV)) of ADP for body fat has been reported to be between 1.7 and 4.5 per cent when measured within 1 day.^31^ Obviously, ADP, as well as hydrostatic weighing, is limited to gross body composition analysis, not making any estimates of regional fat or muscles.

### Bioelectrical impedance analysis (BIA)

BIA uses the electrical properties of the body to estimate the TBW and from that the body fat mass.^32 33^ The body is modeled as five cylindrical LT compartments; the trunk and the four limbs, while fat is considered to be an insulator. The impedance is assumed to be proportional to the height and inversely proportional to the cross-sectional area of each compartment, and the electrical equivalent is a resistor (extracellular water) in parallel with a capacitor and a resistor in series (intracellular water). The model of uniform distribution of fat and water fits better to the extremities than the trunk,^34^ and while there are BIA measurements that correlate well with total abdominal AT, BIA cannot be used for measuring VAT.^35^ Potential error sources are variations in limb length (usually estimated from body height), recent physical activity, nutrition status, tissue temperature and hydration, blood chemistry, ovulation and electrode placement.^32^ BIA requires different model parameters to be used depending on age, gender, level of physical activity, amount of body fat and ethnicity in order to be reliable.^36 37^

### Dual-energy X-ray absorptiometry

DXA is a two-dimensional imaging technique that uses X-rays with two different energies. The attenuation of an X-ray is dependent on the thickness of the tissue and the tissue’s attenuation coefficient, which is dependent on the X-ray energy. By using two different energy levels, the images can be separated into two components (eg, bone and soft tissue). DXA is mainly used for bone mineral density measurements, where it is considered as the gold standard,^38^ but it can also be used to estimate total and regional body fat and LT mass. Pixels, where the ratio between attenuations of the two energies falls below a certain threshold, are classified as soft tissue (ie, without bone), and in those pixels, the attenuation is linearly dependent on the fat fraction of the soft tissue. Pixels above the threshold contain a mixture of bone and soft tissue, and there the soft tissue properties need to be interpolated from surrounding soft tissue pixels.^39^ Approximately one-third of the pixels of the projected body contains bone.^40^

DXA has been found to be more accurate than density-based methods for estimating total body fat.^41^ A possible confounder is that the DXA analysis assumes a constant hydration of lean soft tissue, which is not always true as hydration varies with age, gender and disease.^42^ Excellent repeatability (CV) in the range 1–2 per cent for body fat and 0.5–2 per cent for LT has been reported for DXA.

Since DXA only gives a two-dimensional (coronal) projection, it is not possible to obtain direct compartmental volumetric measurements, so regional volume estimates are obtained indirectly using anatomical models. For example, VAT and parts of the subcutaneous adipose tissue (SAT) are mixed and cannot be separated in the DXA image. The distribution between VAT and SAT then needs to be estimated from an anatomical model predicting the SAT thickness. Furthermore, the physical properties of the technology do not allow for measurements of ectopic fat in organs such as liver fat or muscle fat infiltration. However, due to its ability to estimate regional fat and measure LT, in combination with relatively high availability, DXA has been used for body composition analysis in a wide range of clinical applications.^43^

### CT

CT gives a three-dimensional high-resolution image volume of the complete or selected parts of the body, computed from a large number of X-ray projections of the body from different angles. The known differences in attenuations of X-rays between lean soft tissue and AT can then be used to separate these tissues, as well as to determine mixtures between them. As opposed to the previously described techniques, CT can accurately determine fat in skeletal muscle tissue^16^ and in the liver.^44^ It is, however, significantly less accurate for liver fat <5 per cent which limits its use to diagnose low-grade steatosis.^44^ Being a three-dimensional imaging technique, CT has the potential of giving direct volumetric measurements of organs and different AT depots. In practice, however, CT-based body composition analysis is in most cases limited to two-dimensional analysis of one or a limited number of axial slices of the body, leading to the utilization of the area measured as a proxy for the volume. There are two reasons for this limitation: first, it is important to keep the part of the body being scanned to a minimum in order to minimize the ionizing radiation dose.^45^ This is particularly important in the ethical considerations of research studies on healthy subjects. Second, manual segmentation of different compartments in the images is a very labor intensive task, which can be reduced by limiting the analysis to a few slices rather than a complete three-dimensional volume. This approach, however, limits its precision since the exact locations of slices, in relation to internal organs, cannot be determined a priory and will therefore vary between scans. Nevertheless, CT, together with MRI, is today considered the gold standard for body composition analysis, in particular regional.

### MRI

MRI uses the different magnetic properties of the nuclei of certain chemical elements (normally hydrogen in water and fat) in the cells to produce images of soft tissue in the body. A number of MRI-based methods for quantification of AT (eg, see the review by Hu *et al* 46) and muscles^47–52^ have been developed and implemented in the past.

By using so-called ‘quantitative fat water imaging’, precise measurements of regional AT and LT, as well as diffuse fat infiltration in other organs, can be obtained. The basis for quantitative fat water imaging is fat water separated, or Dixon, imaging,^53^ where the different magnetic resonance frequencies of protons in fat and water are used for separating the two signals into a fat image and a water image. Due to a number of undeterminable factors affecting the MR signal, an MR image is not calibrated on an absolute scale and therefore not quantitative in itself. But by using different postprocessing techniques, the image can be calibrated to quantitatively measure fat or AT. Examples of such methods are proton density fat fraction (PDFF)^54^ measuring the fraction of fat in MR-visible soft tissue and fat-referenced MRI^55–57^ measuring the amount of AT in each voxel.

As opposed to CT and DXA, MRI does not use ionizing radiation, which enables true volumetric three-dimensional imaging even in healthy volunteers and infants. Still, many studies using MRI for body composition analysis have used one or a limited set of two-dimensional slices, mostly due to the lack of efficient image analysis tools for handling three-dimensional image segmentation. However, since there is no ionizing radiation limiting the image acquisition, the slices can be selected from a complete image volume, thereby reducing the uncertainty in their locations. Still, using a sparse set of slices as a proxy for the complete volume will inevitably negatively affect accuracy and precision as only a fraction of the data is used. It has, for example, been shown that single-slice MRI is poor at predicting VAT and SAT changes during weight loss.^58 59^

## BODY COMPOSITION PROFILING USING FAT-REFERENCED MRI

Body composition profiling implies the simultaneous collection and analysis of a number of body composition parameters, including subcutaneous and visceral AT, ectopic fat such as liver and skeletal muscle fat and muscle volumes. Fat-referenced MRI is a methodology that enables all such measurements in one single rapid examination. This section gives a brief introduction to body composition profiling using fat-referenced MRI, together with a review of published validation results of the method. Finally, a previously unpublished validation study of the agreement between this method and DXA for measurements of body fat/AT, body LT and VAT is presented.

The body composition profiling methodology combines fat-referenced MRI with automated image segmentation of different compartments and was first described by Dahlqvist Leinhard *et al.* 55 Different aspects of the method have been further described in other publications.^47 60–62^ The two key features of this method are that it produces quantitative fat-referenced images and that it uses a supervised automated segmentation tool.

In a quantitative fat-referenced image, the value in each image volume element (voxel) represents the amount of fat in that voxel in relation to the amount of fat in pure AT. Hence, a voxel in pure AT has a value of one and a voxel without any fat has the value zero. This means that the following can be measured: the total amount of AT in any given region by summation of the voxel values in that region, AT-free volume by removal of amount of AT from volume measurements of regional LT (eg, muscles) and fractions of fat in specific internal organs, such as the liver.

**Figure 1:**
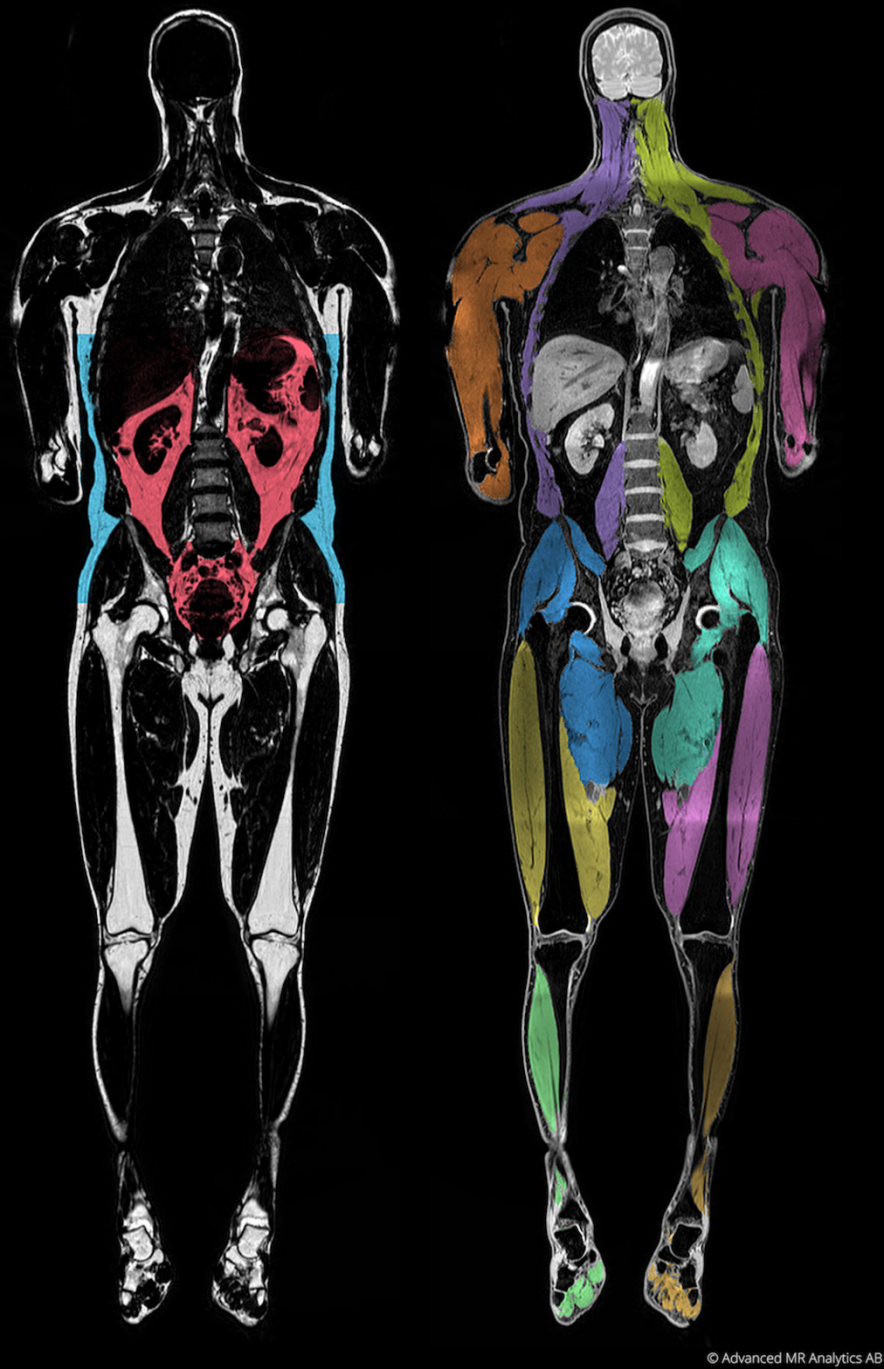
Example of segmentation of abdominal subcutaneous AT (ASAT), visceral AT (VAT) and 10 muscle groups from fat water separated MRI using fat-referenced MRI and multi-atlas image segmentation. To the left is the fat image with ASAT (blue) and VAT (red), and to the right is the water image with the different muscle groups colored. Reproduced with permission from AMRA Medical AB.

The supervised automated segmentation tool enables an efficient way of segmenting different AT compartments, as well as different muscle groups, reducing the manual work to a few minutes, rather than hours, for analyzing a whole-body data set. Anatomical compartments, such as the visceral compartment and different muscle groups, are automatically segmented using predefined anatomical atlases and the operator can then adjust the segmentations if needed. An example of such segmentations is illustrated in figure 1.

See online supplementary appendix 1 for a summary of how fat-referenced MRI is implemented in AMRA Profiler (AMRA Medical AB, Linköping, Sweden), which is the tool for body composition profiling that was used in the validation studies of fat-referenced MRI.

10.1136/jim-2018-000722.supp1 Supplementary data

### Precision and accuracy

In a previous study,^61^ the accuracy of body composition profiling using fat-referenced MRI, in terms of agreement with manual quantification of T1-weighted MR images, was evaluated on 23 (11 females, 12 males) subjects with an average BMI of 31.7±5.1 kg/m^2^ (range 22–46 kg/m^2^); age 36–66 years. There was no significant difference in the measured amount of VAT (4.73±1.99 vs 4.73±1.75 L, P=0.97). Furthermore, the agreement between the methods was excellent for both VAT (95 per cent limits of agreement (LoA) −1.06 to 1.07 L) and abdominal subcutaneous AT (ASAT) (−0.36 to 1.60 L). However, a very small yet statistically significant difference in ASAT was observed (10.39±5.38 vs 9.78±5.36 L, P<0.001). Clearly this small difference has no clinical significance.

Test–retest repeatability and agreement with manual quantification for VAT was evaluated by Newman *et al*.^63^ The study included 30 subjects with five subjects from each gender for each of the following categories of BMI: 18–25 kg/m^2^, 25–30 kg/m^2^ and >30 kg/m^2^. Each subject was scanned twice with at least 20 min interval, during which the subject left the scanner room. There was no significant difference between the evaluated method and manual quantification of VAT (P=0.73). Bland-Altman analysis of the test–retest repeatability showed a bias of −0.04 L (95 per cent LoA −0.12 to 0.13 L) for VAT and 0.05 L (95 per cent LoA −0.55 to 0.64 L) for ASAT. The CV was 1.80 per cent for VAT and 2.98 per cent for ASAT using the method above. The CV for manual quantification of VAT was 6.33 per cent as a comparison.

Middleton *et al* evaluated the accuracy and repeatability of VAT, ASAT and thigh muscle quantification by comparing with manual segmentation on 20 subjects.^64^ Due to the laborious work with manual segmentation, 15 two-dimensional axial slices were manually segmented in the abdominal region for VAT and ASAT and 5 slices over the thigh muscles. For repeatability assessment, the subjects were scanned three times, with the subject remaining in the same position on the scan table between scans 1 and 2 and with the subject removed from the table between scans 2 and 3. The intraexamination (scans 1–2) repeatability test obtained a CV of 3.3 per cent for VAT, 2.2 per cent for ASAT and 1.5 per cent for total thigh muscle volume. For the inter-examination test (scans 2–3), the CVs were 3.6, 2. 6 and 1.5 per cent for VAT, ASAT and thigh muscle volume, respectively. Good agreement with the manual measurements in the 20 slices was observed for all measurements. Neither the slopes nor the intercepts of the regression lines were significantly different from those of the identity lines.

Test–retest repeatability of muscle quantification of left and right abdominal muscles, left and right, anterior and posterior thigh muscles and left and right lower limb muscles, as well as accuracy of lower leg muscle quantification, were evaluated by Thomas *et al* 65 comparing the method above with manual segmentation. The study included 15 subjects of each gender, ranging from normal weight to obese. Each subject was scanned twice with at least 20 min interval, during which the subject left the scanner room. The intraclass correlation (ICC) between the first and second scan was almost perfect (between 0.99 and 1.0) for all muscle groups. The 95 per cent LoA ranged from −0.04 to 0.02 L for the posterior thigh muscles to −0.15 to 0.08 L for the left lower limb. The lowest accuracy for the lower limbs was a bias of −0.08 L with 95 per cent LoA of −0.25 to 0.09 L.

Test–retest repeatability of measurements of VAT and ASAT volumes and volumes and fat infiltration of left and right posterior and anterior thigh muscles, lower leg muscles and abdominal muscles were evaluated by West *et al* on 36 sedentary postmenopausal women.^66^ Each subject was scanned twice, and the subjects were removed from the scanner room between the acquisitions. The intraexamination CV was 1.54 per cent for VAT, 1.06 per cent for ASAT, 0.8–1.9 per cent for volumes of muscle groups (thigh, lower leg and abdomen) and 2.3–7.0 per cent for individual muscle volumes. The 95 per cent LoA was −0.13 to 0.10 L for VAT, −0.38 to 0.29 L for ASAT. The LoA for liver PDFF were within ±1.9 per cent, and for muscle fat infiltration, they were within ±2.06 per cent for muscle groups and within ±5.13 per cent for individual muscles.

The method’s reproducibility of fat-free muscle volume quantification between 1.5 T and 3 T MR scanners, as well as the agreement with manual segmentation, was investigated on 11 different muscle groups.^47^ The ICC between the automated method and manual measurements was at least 0.97 for all muscle groups except in the arms. Except for the arms, the ICC between 1.5 T and 3 T data ranged from 0.97 (left lower leg) to 1.00 (left posterior thigh) with a mean difference volume ranging from 0.39 L (95 per cent LoA 0.01 to 0.77 L) (left abdomen) to 0.0 L (95 per cent LoA −0.10 to 0.09 L) (right lower leg). The muscles of the arms had worse accuracy and reproducibility due to difficulties to include the arms in the field of view.

### Agreement with ADP

A previous study^67^ compared AT measured using fat-referenced MRI with total body fat measured by ADP. The ICC was 0.984. After converting the ADP body fat measures to AT volume (assuming that most of the fat resided in AT and a density of 0.9 kg/L for AT), a Bland-Altman analysis showed that ADP underestimated AT by 0.78 L on average, but the bias was strongly dependent on the level of adiposity with significant underestimation for lean subjects and significant overestimation for subjects with higher amounts of AT. Similar bias dependence has been observed when ADP has been compared with DXA^31^ and MRI.^68^

### Agreement with BIA

Ulbrich *et al* 69 investigated the agreement between fat-referenced MRI and BIA on 80 subjects between 20 and 62 years with a BMI range from 17.5 to 26.2 kg/m^2^. The linear correlation between body fat mass measured by BIA and AT volume measured by MRI was 0.75 and 0.81 for females and males, respectively. The total AT measured by MRI was converted to total fat mass (again assuming that most of the fat resided in AT and using a constant density of 0.94 kg/L). Compared with MRI, the BIA underestimated the total fat with approximately 5 kg (±7 kg LoA) on average, this despite the fact that the MRI-based measurements of total body fat excluded the arms and lower legs. The highest linear correlation found between BIA and MRI-derived measures was 0.75 and 0.81 for females and males, respectively. These correlations were found between BIA-derived body mass percentage and the MRI-derived ‘total AT index’ (total AT divided by body height squared).

### Agreement with DXA

#### Methods and materials

The agreement between DXA and the fat-referenced MRI technique was assessed using data from the UK Biobank study,^70^ approved by the North West Multicenter Research Ethics Committee, UK, and with written informed consent obtained from all subjects prior to study entry. The age range for inclusion was 40–69 years of age. For the present analysis, participants were selected, out of the first 6214 scanned, who had both DXA and MRI scans. One subject with obviously erroneous DXA values (2.7 kg total fat and 6.8 kg LT) was excluded, yielding a total 4753 subjects (2502 females and 2251 males). All included MRI images were analyzable for VAT, ASAT and both thigh muscles according the predefined quality criteria.^62^ The BMI range was 16.4–54.3 with a mean of 26.2 kg/m^2^.

**Figure 2:**
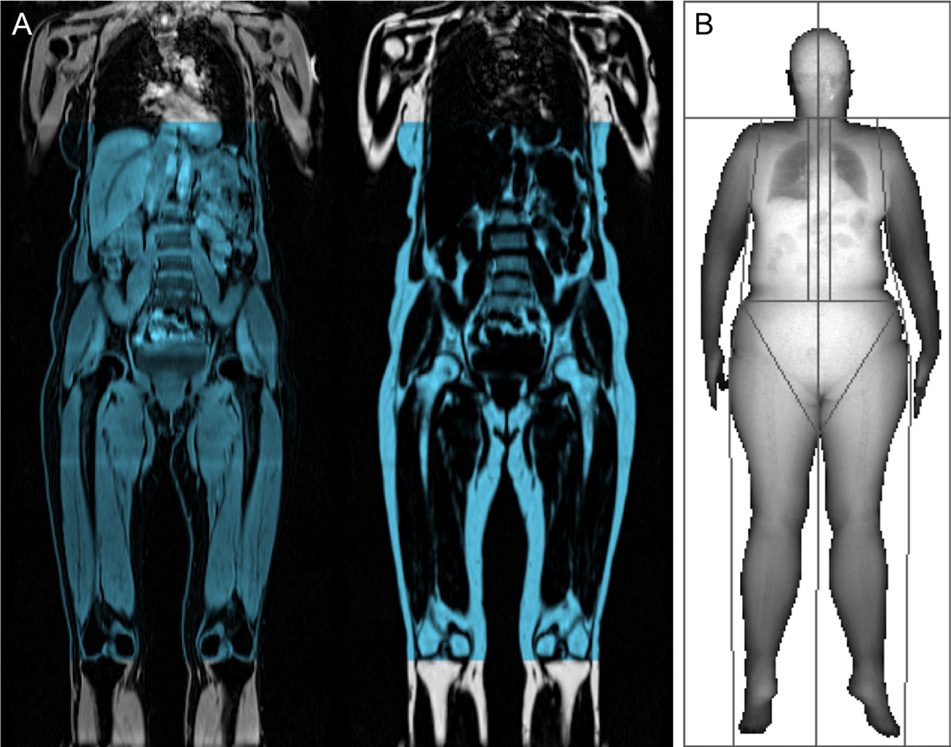
(A) The definition of lean and adipose tissue measured by MRI from the bottom of the thigh muscles to top of vertebrae T9 marked in blue color in the water (left) and fat (right) image. (B) An example of a dual-energy X-ray absorptiometry (DXA) image from the study cohort. DXA image copyright UK Biobank. Reprinted with permission.

The MR images were acquired using a Siemens Aera 1.5 T scanner (Syngo MR D13) (Siemens, Erlangen, Germany) with the dual-echo Dixon Vibe protocol, covering neck to knees as previously described.^62^ The MR images were analyzed using AMRA Profiler. The body AT and LT were measured from the bottom of the thigh muscles to level of the top of vertebrae T9 (figure 2). The LT was defined as the volume of soft tissue subtracted by the volume of AT.^47^

Whole-body DXA data were acquired using a GE-Lunar iDXA (GE Healthcare, Madison, Wisconsin, USA) with the subjects in supine position.^71^ The images were analyzed using the GE enCORE software by the radiographer at, or soon after, the scan. The GE iDXA estimates VAT within an automatically segmented region with the lower border at the top of the iliac crest and its height is set to 20 per cent of the distance from the top of the iliac crest to the base of the skull.^72^

Since the DXA and MRI analyses measure different entities (fat and LT mass vs AT and LT volume, respectively) and they do not cover the same part of the body, a linear model was estimated by linear regression between the MRI and DXA measurements using a training data set of 2376 randomly selected subjects. The remaining 2377 subjects were then used for estimating the agreement between the techniques after linear transformation using the linear model (ie, validating the linear model). The MRI-based measurements (L) were transformed to predict the DXA measurements (kg) using the linear regression coefficients from the training data, and a Bland-Altman analysis was performed to investigate the agreement between MRI-derived and DXA-derived measurements in the validation data. To investigate the agreement between DXA and MRI-derived VAT measurements, a linear model was estimated between the DXA and MRI measurements. Of the 4669 subjects with available DXA VAT measurements, 2334 cases were used to estimate the model and the remaining 2335 subjects were used to validate the agreement between VAT measured by MRI and the transformed DXA measurements using Bland-Altman analysis.

#### Results

**Figure 3:**
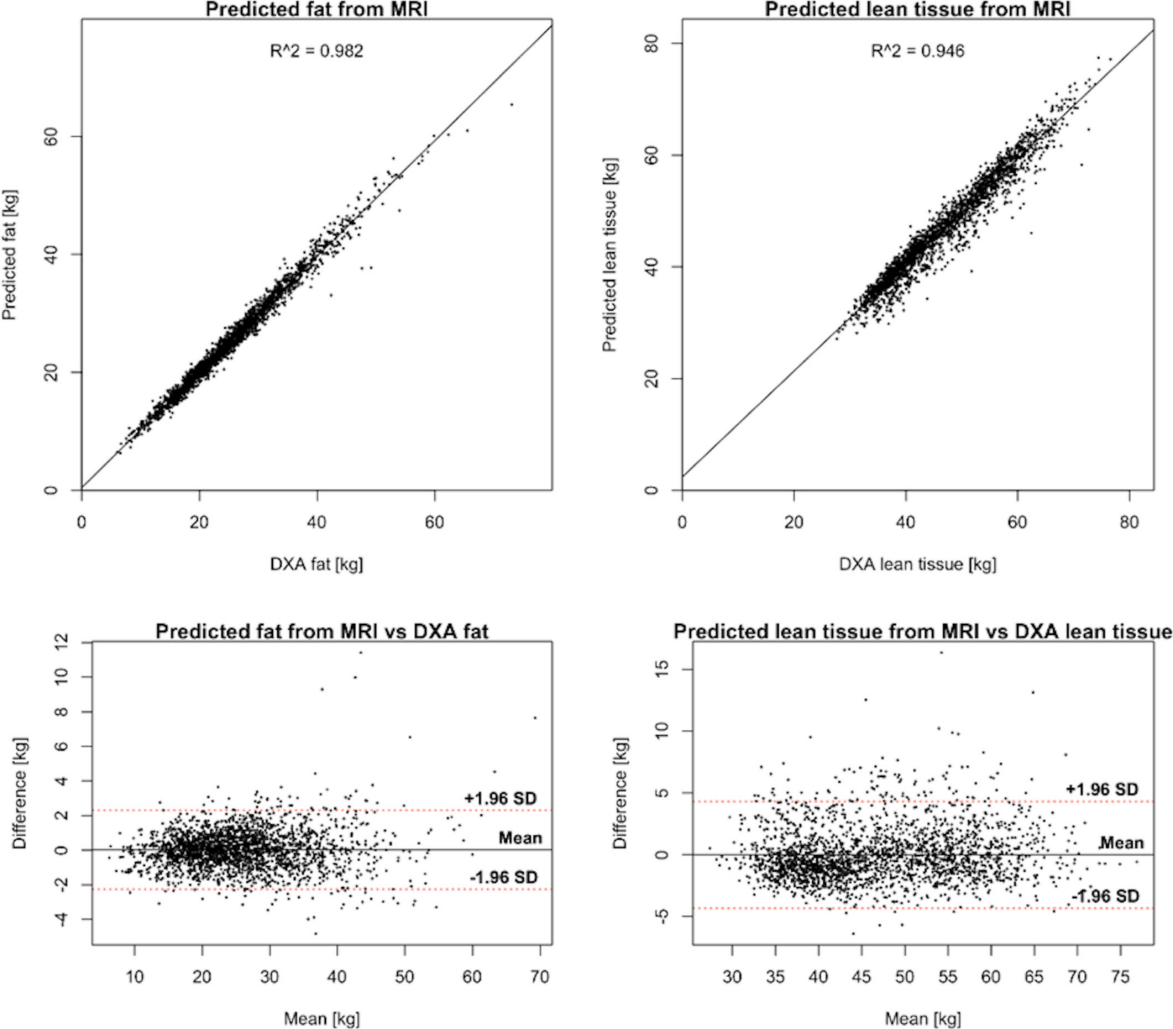
Correlation plots (upper row) between dual-energy X-ray absorptiometry (DXA) and corresponding measurement predicted from MRI using a linear transformation for body fat (left) and body lean tissue (right). The bottom row shows Bland-Altman plots of the agreement between DXA and corresponding measures predicted from MRI.

**Figure 4:**
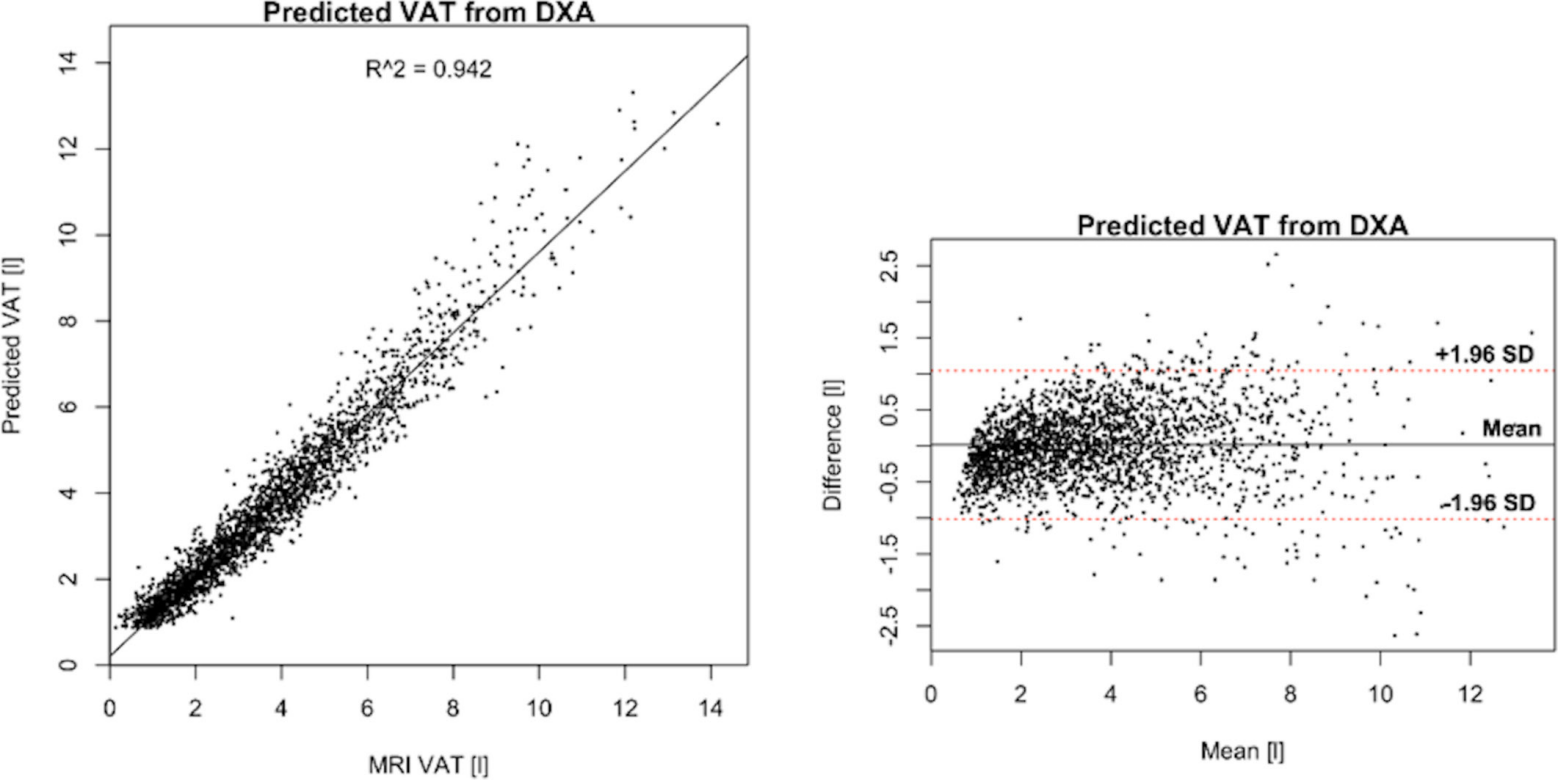
Correlation between visceral adipose tissue (VAT) predicted by dual-energy X-ray absorptiometry (DXA) and VAT measured by MRI (left) and Bland-Altman plot showing the agreement (liters) between the methods (right).

The linear regression between MRI and DXA was 1.23 *x* – 0.12 (kg/L) for body fat/AT and 1.88 *x* + 1.82 (kg/L) for body LT. The linear correlation coefficient, r, between DXA and the transformed MRI measurements was 0.99 for body fat and 0.97 for LT. The 95 per cent LoA from the Bland-Altman analysis were −2.25 to 2.31 kg for fat and −4.33 to 4.31 kg for LT (figure 3). The prediction error SD relative to the mean (CV) was 4.5 per cent for body fat and 4.6 per cent for LT. The correlation between VAT measured by MRI and VAT as predicted by DXA was 0.97 and the LoA were −1.02 to 1.05 L, with CV=21 per cent (figure 4).

## DISCUSSION

Densitometry, including ADP, shows relatively good precision and high correlation with MRI-based measurements of whole-body AT, but with a significant volume-dependent bias. Since these methods only measure the volume or density of the body, they cannot be used for regional measurements and body composition profiling.

BIA is highly available and its relatively low cost is an advantage, which also makes it useful for consumer products. Furthermore, it can differentiate intracellular water from extracellular water, which is a unique capability of BIA. BIA can also, in principle, be used for regional measurements, but it is severely limited when it comes to measuring VAT or ectopic fat in internal organs.

DXA techniques have shown good accuracy when evaluated against MRI for whole-body measurements and very good repeatability. The prediction of whole-body fat and LT from MRI agrees well with DXA after a linear transformation, but less so for VAT. While the correlation between DXA and MRI-derived VAT was high (r=0.97), the agreement after a linear transformation was, however, much lower than for total body fat and body LT, with a CV >20 per cent. The high linear correlation, despite a modest agreement, can be explained by the very wide range of measured VAT volumes, ranging from almost 0 to >14 L. The CV for VAT is in line with the results by Kaul *et al* with a CV of 15.6 per cent for females and 25.9 per cent for males when comparing the same DXA model with CT.^72^ Park *et al* found a linear correlation of 0.85 between VAT measured by DXA and MRI in a study including 90 non-obese men.^73^ However, Kamel *et al* found that the correlation was much lower (r=0.46) for obese men.^74^ The fact that the agreement is lower for obese subjects can also be observed in figure 4 where the prediction error increases with increased VAT volume. Silver *et al* found an excellent correlation without significant bias between fat water MRI and DXA for ‘gross body adipose tissue’ but with a significant negative bias (MRI – DXA) for ‘total trunk adipose tissue’ as well as total and trunk LT.^75^ Interestingly, for DXA, the lowest precision is for fat in the arms, with reported CV up to 11 per cent.^76^ This is the same compartment that is difficult to measure with MRI due to signal loss in the outer parts of the field of view. A strength with DXA, compared with MRI, is the simultaneous assessment of bone mineral density and mass.

When comparing different technologies, both accuracy and precision are important. Accuracy, however, can be rather difficult to compare between technologies for several reasons. First, there is no ground truth available. Even though there is a growing consensus that tomographic methods are the gold standard that can be used to assess accuracy for other methods, they differ between themselves and are difficult to compare in terms of accuracy. Using physical phantoms is one way to assess accuracy, but they miss the difficulties caused by anatomical variations that we know can lead to different measurement errors. Automated tomographic imaging methods can be evaluated against manual methods, but this addresses only one of several important components in the measurement system—the segmentation of different compartments. Second, not all methods measure the same thing, so even if two technologies correlate strongly, there may be a significant bias if they measure different physical entities. For example, AT is not equivalent to fat—besides fat AT also contains water, protein and minerals. When comparing a method that measures AT in volume units, such as MRI, to a method that measures fat in weight units (eg, DXA), we have to convert one unit to the other using a density that is assumed to be constant, which again may not be always accurate.

Although this review has not focused on measurements of ectopic fat, this is an important component in body composition profiling, especially for understanding metabolic status and assessing risk. Among the techniques discussed here, CT and quantitative MRI are the only methods that can quantify local diffuse infiltration of AT and ectopic fat. (Non-invasive measurements of ectopic fat, in particular liver fat, are commonly done by MR spectroscopy (MRS), but since MRS only measures local substance concentrations and not absolute amounts of fat, AT or LT, this technology was not included in this study.) While it is possible—and sometimes necessary—to use different equipment for different measurements in a study, it is often desirable to keep the number of different examinations and modalities to a minimum in order to optimize the work flow. By using quantitative MRI, or CT if the radiation dose is not a concern, a large number of metabolically relevant body composition parameters can be measured with high accuracy and precision in a single examination.

A comparison of the capabilities of different measurements of the techniques discussed above is summarized in table 1.

**Table 1:** Comparison of the capabilities of different techniques for body composition analysis

	ADP	BIA	DXA	CT	MRI
Total fat	Yes	Yes	Yes	Yes	Yes
Total lean tissue	Yes	Yes	Yes	Yes	Yes
VAT	No	No	Approximate	Yes	Yes
Volume of individual muscles	No	No	No	Yes	Yes
Diffuse fat infiltration	No	No	No	Yes	Yes
Without ionizing radiation	Yes	Yes	No (low)	No	Yes

ADP, air displacement plethysmography; BIA, bioelectrical impedance analysis; DXA, dual-energy X-ray absorptiometry; VAT, visceral adipose tissue.

## CONCLUSION

There are several methods available that can measure whole-body AT or fat and LT. In terms of precision and accuracy, DXA and MRI are comparable as they show excellent agreement after a linear transformation. However, the agreement is much lower for compartmental measurements such as VAT. Moreover, MRI gives access to accurate and direct measurements of diffuse infiltration of AT in muscles and ectopic fat (eg, liver fat). Rapid MRI scanning protocols, in combination with efficient image analysis methods, have promoted MRI to a competitive option for advanced body composition assessment, thus enabling a more complete description of a person’s body composition profile from a single examination.

10.1136/jim-2018-000722.supp2 Supplementary data
