# Supplementary material for: Advanced body composition assessment: from body mass index to body composition profiling
Source: J Investig Med. 2018 Mar 25;66(5):1–9. doi: 10.1136/jim-2018-000722 (PMC5992366; doi:10.1136/jim-2018-000722)
Supplement: Supplementary data [file jim-2018-000722supp001.pdf]

Appendix 1

MR Image Analysis Method for Body Composition Profiling

The analysis method comprises the following steps:

1. Fat-water separated images are collected using a rapid two-point Dixon protocol using a 3D gradient-echo protocol with a number of stations, together covering at least neck to knees. Normally, a neck-to-knee scan takes approximately 6 minutes[1].
2. The fat- and water-separated image volumes are calibrated using the fat signal in pure AT as signal reference[2 3].
3. The calibrated volumes are automatically merged to one volume.
4. A soft tissue mask (STM) is computed by first crating a binary tissue mask by thresholding the sum of the calibrated fat and water signals at 0.5. Voxels within one voxel from the borders of the binary tissue mask are set equal to the sum of the normalized water and fat images to account for partial volumes at the tissue interface.
5. Regions containing VAT and abdominal subcutaneous tissue (ASAT) are segmented using non-rigid multi-atlas-based segmentation[2]. VAT is here defined as the AT within the abdominal cavity, excluding AT outside the abdominal skeletal muscles and AT and lipids within and posterior of the spine and posterior of the back muscles. ASAT is defined as subcutaneous AT in the abdomen from the top of the femoral head to the top of the thoracic vertebrae T9. Also, the larger muscle groups are segmented using the same approach[4]. An example of such segmentations is illustrated in Figure 1.
6. The segmented regions are visually inspected by a trained analysis engineer. The operator can interactively adjust the final segmentation in subjects with atypical anatomies.
7. VAT and ASAT volumes are quantified by integrating the calibrated fat image multiplied by the STM within the segmented regions respectively and then multiplying with the voxel volume.
8. Lean tissue can be measured in arbitrary regions of interest (ROI) by multiplying the binary ROI mask with the STM and subtract the fat signal with the mask and finally multiplying with the voxel volume.
9. "Fat-free" muscle volume (i.e. the volume of the muscle after removing most of the AT within the segmented muscle) is measured for different muscle groups by measuring the volume of the segmented muscle group multiplied by the STM and subtracted by the volume of voxels containing more than 50 % AT[4].
10. Diffuse infiltration (%) of AT (and also intra-myocellular fat) in muscles is measured by integrating the calibrated fat image under the STM and then divide this by the muscle volume.
11. Liver fat (%) can be measured using PDFF in a separate scan covering the liver or using the fat-referenced calibrated image after adjustment for PDFF in AT.

The fat-referenced calibration (step 2) is a key feature, in which the MR fat signal from pure AT is interpolated over the image and used as reference compensating for inhomogeneities in the imaging system. The result is a calibrated fat image, in which pure AT has (approximately) the value one. Hence, the intensity value in each voxel corresponds to the amount of AT within that voxel. This makes the analysis robust with respect to partial volume effects due to limited image resolution and also enables measurement of diffuse AT infiltration in muscles and ectopic fat in other organs such as the liver. The fat-referenced calibration makes the fat image robust against T1 bias, thereby enabling a rapid T1-weghted scan while maintaining a high signal-to-noise ratio[5].

References

1. West J, Dahlqvist Leinhard O, Romu T, et al. Feasibility of MR-Based Body Composition Analysis in Large Scale Population Studies. *PLOS ONE* 2016;11(9):e0163332. doi: 10.1371/journal.pone.0163332
2. Dahlqvist Leinhard O, Johansson A, Rydell J, et al. Quantitative Abdominal Fat Estimation Using MRI. *INTERNATIONAL CONFERENCE ON PATTERN RECOGNITION* 2008;CONF 19:2137-40.
3. Romu T, Borga M, Dahlqvist Leinhard O. MANA - Multi scale adaptive normalized averaging. 2011 IEEE International Symposium on Biomedical Imaging: From Nano to Macro: IEEE conference proceedings, 2011:361-64.
4. Karlsson A, Romu T, Borga M, et al. Automatic and quantitative assessment of regional muscle volume by multi-atlas segmentation using whole-body water-fat MRI. *Journal of Magnetic Resonance Imaging* 2015;41(6):1558-69. doi: 10.1002/jmri.24726
5. Peterson P, Romu T, Brorson H, et al. Fat quantification in skeletal muscle using multigradient-echo imaging: Comparison of fat and water references. *Journal of Magnetic Resonance Imaging* 2016;43(1):203-12. doi: 10.1002/jmri.24972
